# Isotopic resonance at 370 ppm deuterium negatively affects kinetics of luciferin oxidation by luciferase

**DOI:** 10.1038/s41598-018-34704-0

**Published:** 2018-11-02

**Authors:** Sergey Rodin, Paola Rebellato, Arne Lundin, Roman A. Zubarev

**Affiliations:** 1 Division of Physiological Chemistry I, Department of Medical Biochemistry and Biophysics, Karolinska Institutet, Scheelesväg 2, SE-17 177 Stockholm, Sweden; 2 Biothema AB, Handens stationsväg 17, 136 40 Handen, Sweden; 3 Department of Pharmacological & Technological Chemistry, I.M. Sechenov First Moscow State Medical University, Moscow, Russia; 4 Cardiothoracic Research Center, Department of Surgical Sciences, Uppsala University, Regementsvägen 1, 752 37 Uppsala, Sweden

## Abstract

Since 1930s, it has been known that some biochemical and biological processes exhibit abnormal kinetics at a deuterium concentration in the local environment of 250–600 ppm, which is 2–4 times higher that the normal concentration of 150 ppm D. We sought to test if the kinetics of firefly luciferase oxidizing luciferin, the reaction widely used as a read-out in various biochemical assays, is also affected by an elevated deuterium content. To this end, both luciferase and luciferin substrate solutions were prepared based on water with extra deuterium added to a concentration ranging from 150 ppm and up to 10,000 ppm (1%). Upon mixing the solutions, the luminescence intensity at different times was compared with that of the corresponding control solutions with 150 ppm D. A broad negative resonance was detected (p < 10^−6^), with a ≈20% drop in luminescence at 370 ppm D. Given that, on average, about half of hydrogen atoms in proteins are not exchangeable in solution, this value corresponds to ≈260 ppm of deuterium in all enzyme’s hydrogens, in a very good agreement with the prediction of the Isotopic resonance hypothesis.

## Introduction

Since 1930s it has been known that water slightly enriched with deuterium (to 250–600 ppm D compared to the normal value of 150 ppm D) had a marked effect on various organisms as well as certain enzymes^1–11^. While growth of biological organisms was typically accelerated, the digestion of starch by pancreatic amylase and the fermentation of glucose by zymin (enzyme mixture extracted from germinating seeds) were found to be retarded^5^. Importantly, the retardation of hydrolysis by 10–15% was only obtained when the enzyme had been exposed to the isotopically enriched water for sufficiently long time (≥16 h). The effect was absent when the substrate was incubated in place of the enzyme.

Following the same line of research with more advanced methods, Lobyshev *et al*. have found in 1970s that the Na, K-ATPase activity increases at elevated deuterium concentrations by up to 50%, reaching maximum at 400–500 ppm D^7,8^.

Using modern instrumentation and methodology, here we sought to test if the kinetics of firefly luciferase oxidizing luciferin is also sensitive to low-level enrichment of deuterium in water. Luciferase is an enzyme that generates light when acts on the luciferin substrate. Photon emission can be detected by light sensitive equipment, thus allowing for direct observation of biological processes. Luciferase activity is often used as a reporter read-out to assess the kinetics of other enzymes or other types of biological activity both in research and industry^12^. It is also used in ATP-based detection of bacteria in water, beverage and food, with the detection limit of 1 attomole corresponding to the amount of ATP in a single bacterial cell^13^. Thus the effect of deuterium concentration on luciferase + luciferin kinetics is of potential interest in a variety of applications. Due to extensive use in chemometry, the luciferase assays are very well characterized and standardized; therefore, they represent a reliable test object for studying kinetic effects of various nature.

## Results

### Experiment 1. Time-course of luciferase activity in 150, 300, and 10,000 ppm of deuterium

**Figure 1:**
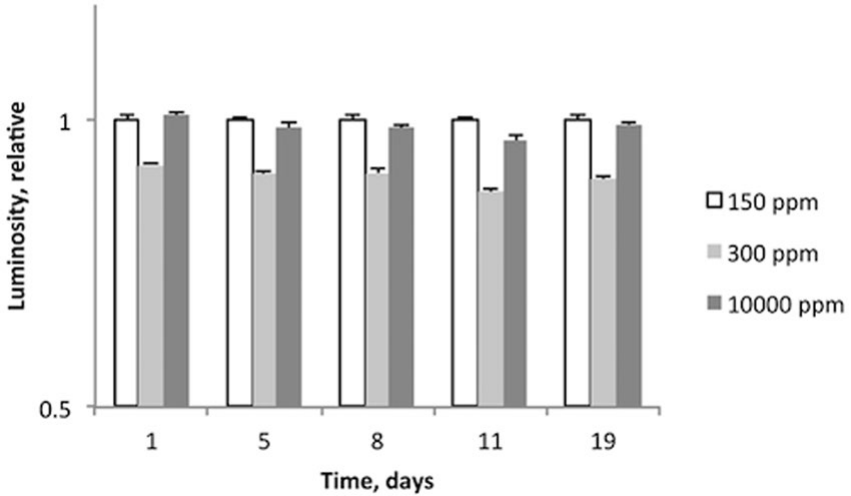
Relative intensities of Luciferase luminescence measured on the first, firth, eight, eleventh, and nineteenth days after reconstitution of luciferase solution and luciferin substrate with solutions containing 150, 300, and 10 000 ppm D. ATP was reconstituted with 300 ppm D water in all cases. Error bars represent standard deviation.

In order to determine how the time passed after the reconstitution of luciferase solution and luciferin substrate solution affected the measurements, the light intensities were measured after two hours on the first day, and on the fifth, eighth, eleventh, and nineteenth days. The intensity of luminescence in the cuvettes with 300 ppm D was always 10–15% lower than in the cuvettes with 150 and 10,000 ppm of deuterium (Fig. 1), with no significant difference between the 150 ppm D and 10,000 ppm D cuvettes. A slight downward drift with time was observed in all groups, but the ratio between the signals at different deuterium concentrations remained largely the same for all time points.

### Experiment 2. Verification of the reduction in luciferase activity at 300 ppm

**Figure 2:**
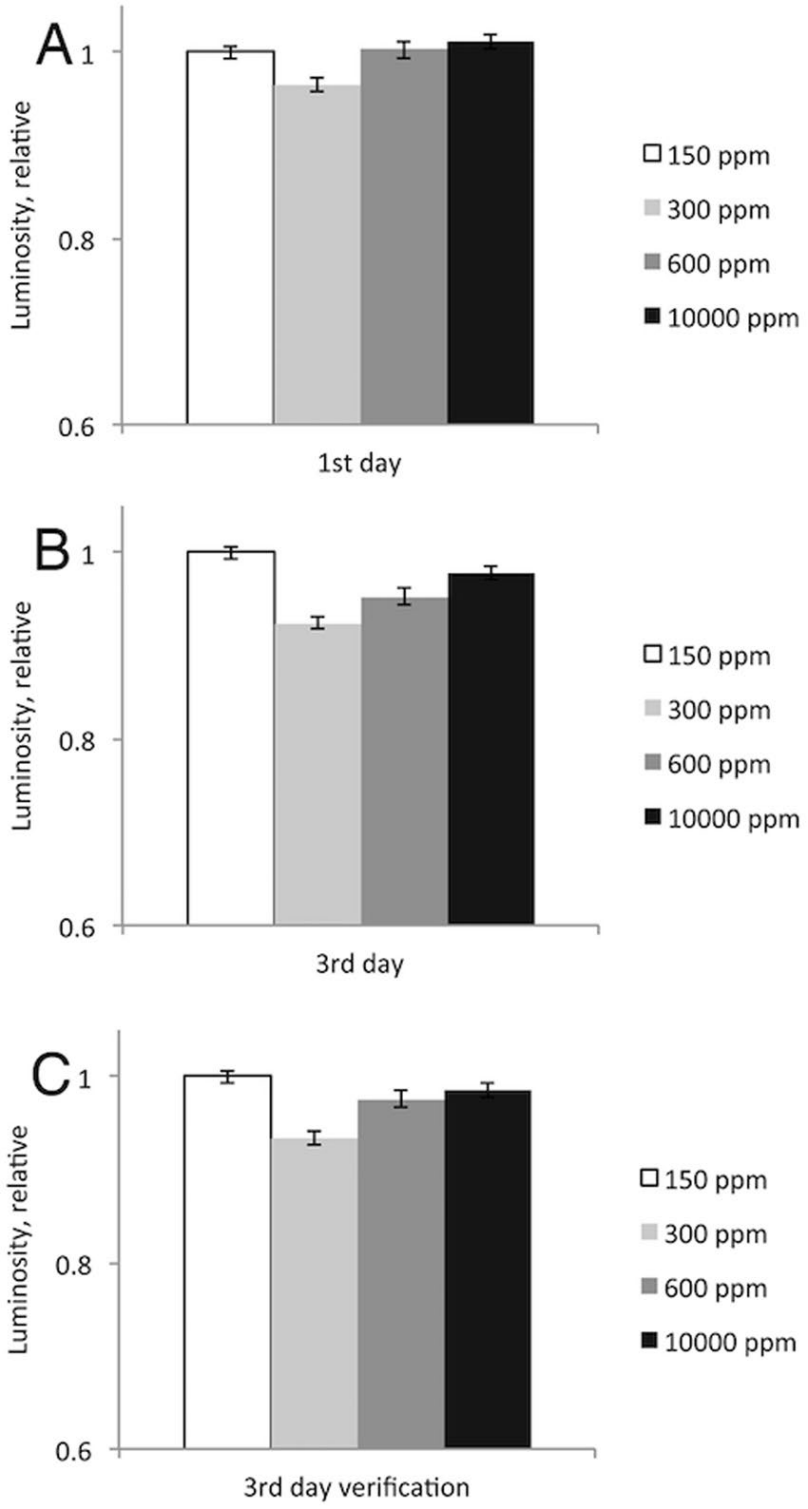
Relative intensities of Luciferase luminescence measured on various days after reconstitution of luciferase solution and luciferin substrate with solutions containing 150, 300, 600, and 10000 ppm D. (**A**) On the first day after the reconstitution. ATP was reconstituted with 300 ppm D water. (**B**) On the third day after the reconstitution. ATP was reconstituted with 300 ppm D water. (**C**) On the third day after the reconstitution. ATP was reconstituted with 150 ppm D water. Error bars represent standard deviation.

To verify and further investigate the decrease in luciferase activity observed at 300 ppm D in experiment 1, all solutions were prepared anew and the luminescence intensities were measured for 150, 300, 600, and 10,000 ppm D on the first and the third day after reconstitution of luciferase and luciferin substrate solutions, with ATP solution reconstituted with water containing 300 ppm D (Fig. 2A and B). Confirming our previous finding, the luminescence at 300 ppm D was ≈10% lower than that at 150 ppm D and 10,000 ppm D, with 600 ppm D showing an intermediate result.

To test if the reconstitution of ATP with 300 ppm D water affected the outcome of the experiment, the same measurements were performed using ATP reconstituted with 150 ppm D water. The results were nearly identical (Fig. 2C). Therefore, the observed effect was mainly due to the deuterium content in water used for reconstitution of the enzyme and luciferin, but not ATP.

### Experiment 3. Detailed mapping the luciferase + luciferin kinetics in the range between 150 and 600 ppm D

**Figure 3:**
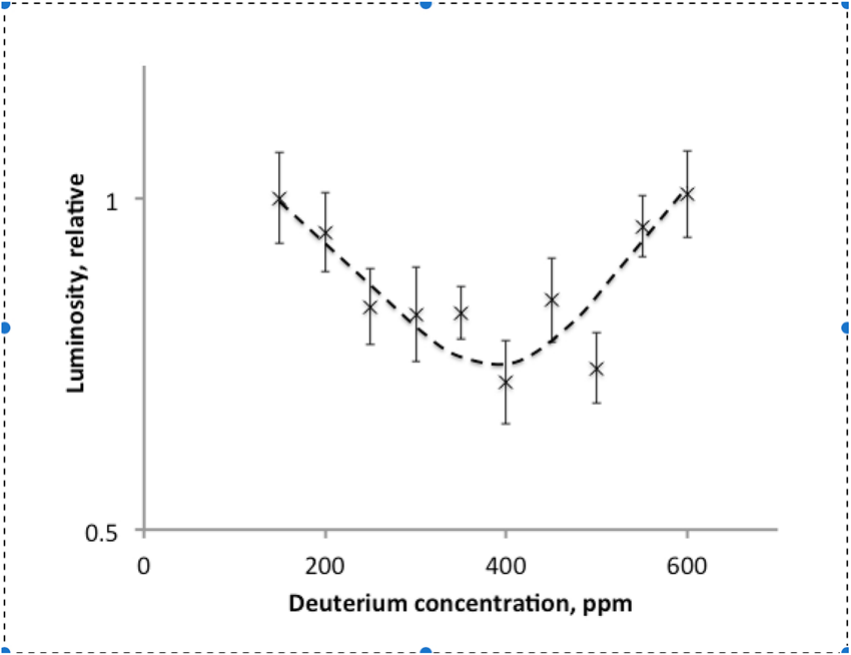
Relative intensities of Luciferase luminescence measured on the third day after reconstitution of luciferase solution and luciferin substrate with solutions containing 150, 200, 250, 300, 350, 400, 450, 500, 550 and 600 ppm D. ATP was reconstituted with 300 ppm D water. Error bars represent standard deviation.

To precisely map the area of deuterium concentrations with abnormal luciferase activity, 10 concentrations of deuterium in the range between 150 and 600 ppm D were tested for luciferase and luciferase substrate solutions. The luminescence intensities were significantly lower in cuvettes with deuterium content between 250 and 500 ppm D, while the luminescence at 150 ppm was nearly identical to that at 600 ppm D (Fig. 3). A broad negative resonance with a 20% lower luminescence was detected with high statistical power (p < 10^−6^). The center of gravity calculations for the plot in Fig. 3 reveals that the peak of the negative resonance is located at 370 ± 10 ppm D.

## Discussion

The existence of a resonance (positive or negative) in the region of deuterium concentrations from 250 ppm to 600 ppm has been found for a number of biological processes, but mostly at the level of whole cells or organisms^1–11^. Since even a simple biological species, such as bacterium, is an extremely complicated biochemical system whose growth involves thousands of reactions, of particular interest is to determine how the deuterium content affects kinetics of individual enzymatic reactions. In that respect, luciferase + luciferin represents an important model system. Due to the ease with which its kinetics can be measured with high sensitivity and accuracy, these measurements are widely used in biology.

Here we found that the luciferase activity in oxidizing luciferin decreases by ca. 20% at 370 ppm D. This value of deuterium concentration is in excellent agreement with the positive activity spike observed by Lobyshev *et al*. for Na,K-ATPase activity^6,7^. As in Lobyshev’s experiments, the deuterium effect on luciferase activity disappeared above ≈600 ppm D, and even much larger deuterium concentration of 10,000 ppm resulted in a significantly smaller (<5%) activity reduction.

At 150 ppm D, there is only one deuterium atom for every 6000 hydrogen atoms in the system. From the point of view of conventional chemical kinetics paradigm, doubling deuterium content should produce only a miniscule effect. To the best of our knowledge, the only scientific model capable of explaining the above results and predicting a significant kinetics change at concentrations of 250–350 ppm D is that of the isotopic resonance (IsoRes)^14–17^. In brief, the IsoRes model postulates that at certain “resonance” abundances of stable isotopes, a certain compositional symmetry emerges, which reduces the complexity of the system built of these elements. This complexity reduction affects the kinetics of both the direct and reversed reactions within the system. This, in turn, may shift the equilibrium either way. Many chemical reactions increase their kinetics, but some (e.g., starch hydrolysis^5^) decrease their rate.

The fact that the average terrestrial isotopic compositions for the elements C, H, N and O are close to a resonance for proteins has likely contributed to the origin of life on Earth^14^. The IsoRes model predicts that this terrestrial resonance can be made even stronger by roughly doubling the natural deuterium content, with the isotopic abundances of other elements remaining the same^13,15^. The is therefore that, at deuterium content of 250–350 ppm D (depending upon the exact value of the isotopic abundances of other elements), the rate of many biochemical reactions should change, returning back to the normal levels at ≥600 ppm D.

It is worth noting that roughly half of the hydrogen atoms in proteins (those connected to other atoms than carbon) are easily exchangeable in solution. Therefore, placing a protein expressed at 150 ppm D into a 360 ppm D solution results eventually in *average* deuterium content in the protein of ≈260 ppm. This value is close to the optimal deuterium concentration predicted by the IsoRes model. The model however is incapable at the moment to predict the sign of the resonance, i.e., whether the kinetics will accelerate or slow down. Examples from literature and our own experience show that in a majority of cases, an acceleration is observed, but in some instances, as in the abovementioned starch hydrolysis, the effect of an IsoRes on kinetics is negative.

As was first reported by Barnes^5^, for reliable observation of kinetic effects it is important to achieve a quasi-equilibrium state by incubating enzymes in solution with altered deuterium content for extended period of time. Barnes used two weeks of incubation. He noticed that the enzymes freshly dissolved in a solution with certain H/D composition may not necessarily exhibit the phenomenon, or the effect magnitude may widely fluctuate around lower than optimal value, spoiling the statistical certainty of the observations. This may explain why in our experiments, the largest magnitude was observed in the last experiment, where the thermally-assisted water mixing was the most thorough. Curiously, sophisticated X-ray emission spectroscopy revealed that water and alcohol don’t mix at molecular level even after very long incubation times^18^. This finding supports the need for very careful and long mixing of water-based solutions.

As a final comment, the isotopic resonance effects can be observed not only for deuterium but also for other stable isotopes. Recently, the predicted IsoRes at ≈3.5% ^15^N^6^ was independently confirmed in two organisms^19,20^.

## Online Methods

### Preparation of water and TRIS-EDTA buffer with elevated deuterium concentrations

Milli-Q grade water (150 ppm D) and TRIS-EDTA buffer (Cat. N 21-103; BioThema AB, Sweden) were spiked with 98% D_2_O (Sigma) to achieve deuterium concentrations up to 10,000 ppm D (1% D). Control solutions (150 ppm D) were spiked with the same volume of Milli-Q water. The solutions were mixed for 2 days on a rocker at room temperature (≈22 °C). After that, the solutions were subjected to warming-cooling cycles with warming till 70 °C and cooling down to room temperature, to ensure more homogeneous distribution of deuterium. Solutions for experiments 1 and 2 were subjected to 3 heating/cooling cycles and for experiment 3 - to 5 cycles. After that, the solutions were additionally mixed using a rocker for one day at room temperature.

### Preparation of solutions for luciferase activity measurements

ATP reagent SL (Cat. N 11-501; BioThema AB, Sweden) containing recombinant luciferase from the Japanese firefly *Luciola cruciata* (Kikkoman, Chiba, Japan), luciferin, magnesium sulphate and stabilizers but not ATP was reconstituted in 10 mL of water with various deuterium concentrations. This solution was used as a source of luciferase. Luciferin Substrate (Cat. N 61-101; BioThema AB, Sweden) was reconstituted in 1 mL of water with various deuterium concentrations. 5 µL of the ATP reagent SL solution was added into 1 mL of the Luciferin Substrate. The obtained Luciferase plus Luciferin solution was stored at +4 °C and was allowed to reach room temperature shortly before measurements. Immediately before the experiments, ATP Substrate (Cat. N 51-101; BioThema AB, Sweden) was reconstituted in 0.96 mL of 300 ppm deuterium water except for confirmation measurements in Experiment 2, where ATP Substrate was reconstituted in a 150 ppm D water.

### Measurements of luciferase activity

Luminometer 1251 (BioOrbit, Turku, Finland) with the capacity to measure in one run the light intensity from up to 25 cuvettes (4 mL each; Sarstedt, Germany) was used. Samples were placed in the luminometer in chess order to reduce the influence of the position. Three groups of 8 cuvettes each occupied 24 slots and the 25^th^ cuvette was always used as a blank control to determine the baseline. Six or eight cuvettes per each concentration of deuterium were measured at a time, thus comparing three or four sample/control groups, respectively. In experiment 3, nine samples representing various concentrations of deuterium were divided into three groups, each group containing a control sample with 150 ppm D to normalize the signals.

For each cuvette, 0.41 mL of TRIS-EDTA buffer with certain concentration of deuterium was mixed with 50 µL of the Luciferase plus Luciferin solution with the same deuterium concentration. Then 40 µL of ATP Substrate solution containing 300 ppm of deuterium were added by the dispenser of the luminometer (the same solution to all 25 cuvettes). Since 40 µL represented only 8% of the whole reaction volume, the overall concentration of deuterium in a given sample did not changed significantly.

Luminesce intensity was measured at 25 °C according to a standard procedure (Supplementary Materials). The luminesce counts were imported into Excel program, grouped, normalized, with mean values and standard deviations calculated.

## Electronic supplementary material

Supplementary Dataset 1
